# Supplementary figures and images for: A Single-Cell Atlas of Porcine Skeletal Muscle Reveals Mechanisms That Regulate Intramuscular Adipogenesis
Source: Int J Mol Sci. 2024 Dec 1;25(23):12935. doi: 10.3390/ijms252312935 (PMC11641529; doi:10.3390/ijms252312935)

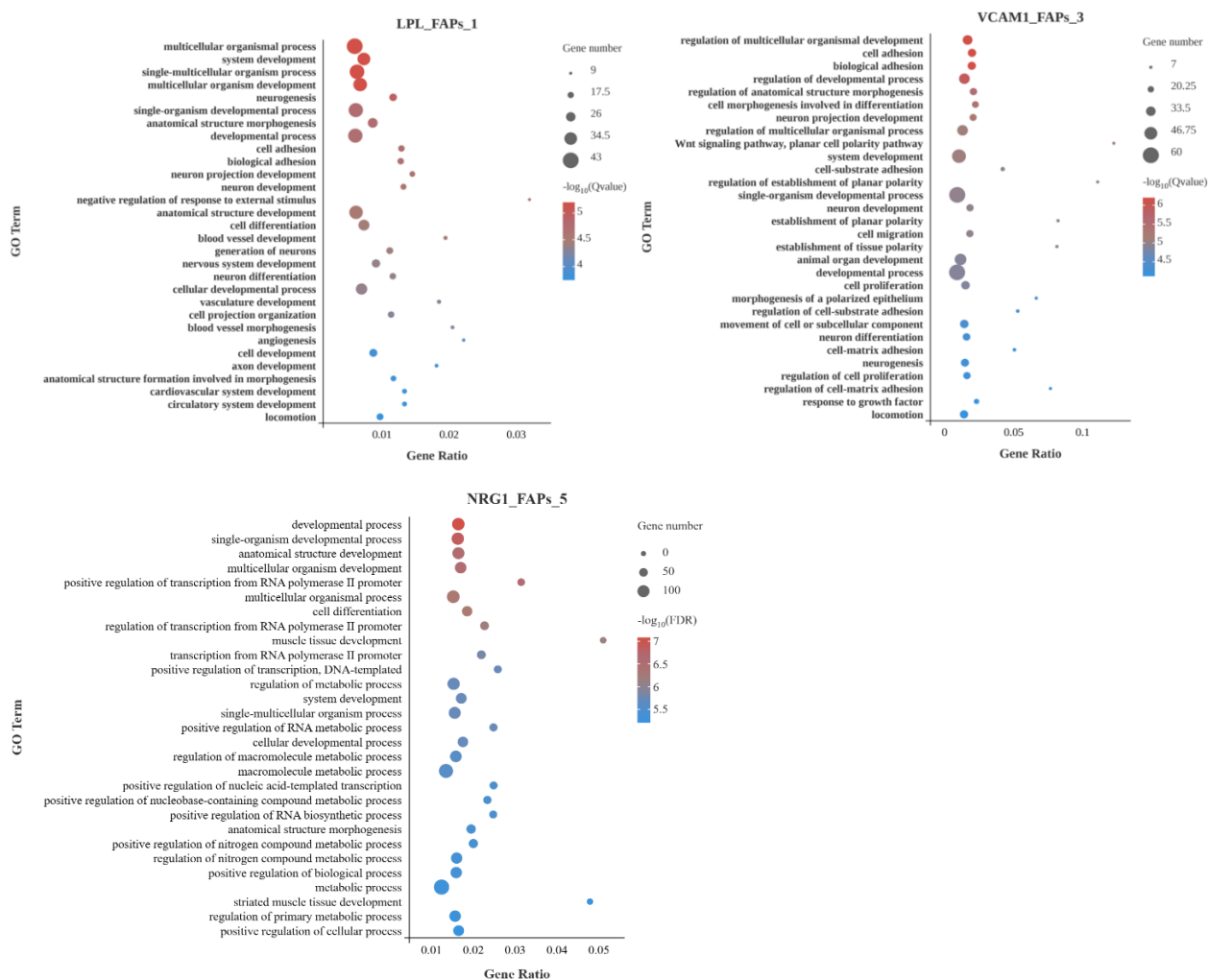

**Figure S1.** GOBP enrichment analysis of FAP 1, 3, and 5 genes.

Supplement: Supplementary file 1 [file ijms-25-12935-s001.zip › Figure S1.pdf]
